# Models incorporating physical, laboratory and gut metabolite markers can be used to predict severe hepatic steatosis in MAFLD patients

**DOI:** 10.1002/kjm2.12904

**Published:** 2024-11-04

**Authors:** Yi‐Hsuan Lin, Ching‐Hsiang Wang, Yen‐Hsun Huang, Hsiao‐Chin Shen, Wei‐Kai Wu, Hsiao‐Yun Yeh, Chia‐Chang Huang, Chien‐Wei Su, Ying‐Ying Yang, Ming‐Shiang Wu, Han‐Chieh Lin, Ming‐Chih Hou

**Affiliations:** ^1^ Department of Medical Education Taipei Veterans General Hospital Taipei Taiwan; ^2^ School of Medicine, College of Medicine, National Yang Ming Chiao Tung University Taipei Taiwan; ^3^ Department of Family Medicine Taipei Veterans General Hospital Taipei Taiwan; ^4^ Bachelor Program of Biotechnology and Food Nutrition National Taiwan University Taipei Taiwan; ^5^ Department of Medical Research National Taiwan University Hospital Taipei Taiwan; ^6^ Division of General Medicine, Department of Medicine Taipei Veterans General Hospital Taipei Taiwan; ^7^ Institute of Clinical Medicine, School of Medicine, National Yang Ming Chiao Tung University, Taipei, Taiwan Taipei Taiwan; ^8^ Division of Gastroenterology and Hepatology, Department of Internal Medicine Taipei Veterans General Hospital Taipei Taiwan; ^9^ Department of Internal Medicine National Taiwan University Hospital Taipei Taiwan

**Keywords:** fatty liver, metabolic‐associated fatty liver disease, short‐chain fatty acids, tryptophan

## Abstract

Metabolic‐associated fatty liver disease (MAFLD) induced‐severe hepatic steatosis poses significant health risks. Early prediction of this condition is crucial for prompt intervention. Short‐chain fatty acids (SCFAs) and tryptophan are gut metabolites correlated with MAFLD pathogenesis in the gut–liver axis. This study aims to construct prediction models for severe hepatic steatosis by including SCFAs and tryptophan metabolites. This study enrolled 83 participants from the outpatient department in 2023. Physical measurements, serum metabolic and inflammatory markers, metabolites of serum SCFAs and tryptophan were collected. Severe hepatic steatosis was diagnosed using vibration‐controlled transient elastography and abdominal sonography. All 40 (48.2%) participants diagnosed with severe hepatic steatosis had MAFLD, while approximately three‐quarters of those without severe hepatic steatosis had MAFLD. In comparison to the non‐severe hepatic steatosis group, individuals with severe hepatic steatosis exhibited higher levels of waist and arm circumference, serum triglyceride (TG), and lower levels of serum high‐density lipoprotein cholesterol (HDL‐C) and AST/ALT ratio. They also had higher serum levels of lipopolysaccharide‐binding protein, isovaleric acid, and propionic acid, and lower levels of 3‐methylvaleric acid, indole‐3‐propionic acid, and indoxyl sulfate. Models incorporating these markers predicted severe hepatic steatosis. One model additionally included waist circumference and triglyceride‐glucose index, while the other incorporated arm circumference and TG/HDL‐C ratio. The area under the curve reached 0.958 and 0.938, respectively (*p* < 0.001). SCFAs and tryptophan metabolites are valuable in predicting severe hepatic steatosis. Further research is needed to investigate the roles of these metabolites in MAFLD.

## 1 INTRODUCTION

Metabolic‐associated fatty liver disease (MAFLD) is the most popular chronic liver disease nowadays and its prevalence is still increasing.^1^ About one‐quarter of MAFLD patients progress to hepatitis, which further increases the risk of cirrhosis and hepatocellular carcinoma.^2^ Patients with more fat accumulated in the liver are more susceptible to advanced liver diseases.^3^ Therefore, identifying severe hepatic steatosis is important to enable prompt management and avoid progression to serious consequences.

The pathogenesis of MAFLD is a multifactorial and interconnected process.^4^ Dysbiosis and systemic inflammation increase intestinal permeability, elevating the level of serum lipopolysaccharide‐binding protein (LBP), which further exacerbate systemic inflammation via the gut–liver axis.^5^ Dysbiosis also affects gut metabolites, such as short chain fatty acids (SCFAs) and tryptophan. Thing et al. ever reported higher plasma levels of propionate, valerate, α‐methylbutyrate, and a lower level of acetate in MAFLD patients.^6^ In tryptophan metabolism, inflammatory cytokines increase kynurenine production and exacerbate steatosis,^7^ while indole may suppress inflammatory cytokines and alleviate liver inflammation.^8^ Despite these studies, literature about metabolites of SCFAs and tryptophan in MAFLD is limited.

The vibration‐controlled transient elastography (VCTE) is a practical tool used to diagnose steatosis. VCTE outputs the value of controlled attenuation parameter (CAP) used to quantify the fat content in liver and is proved to be accurate in detecting hepatic steatosis.^9^ While VCTE stands as a reliable diagnostic tool for hepatic steatosis, the development of prediction models is a potentially cost‐effective alternative and can serve as a screening method within primary care settings. Many studies tried to predict MAFLD by using clinical variables.^10^, ^11^ Nevertheless, whether incorporating factors relevant to the gut–liver axis and gut metabolites in the prediction models increases the accuracy of predicting MAFLD is unknown.

This study aims to develop prediction models for severe hepatic steatosis by incorporating SCFAs, tryptophan metabolites, and other important variables relevant to the pathomechanism of MAFLD. Having precise prediction models will enable healthcare professionals to identify severe hepatic steatosis early and initiate appropriate treatment strategies promptly.

## 2 MATERIALS AND METHODS

### 2.1 Study design

This cross‐sectional study is the first‐wave analysis of research projects on MAFLD at Taipei Veterans General Hospital (VGHTPE), Taipei, Taiwan. We recruited participants from outpatient clinic of general internal medicine from February 2023 to December 2023. Adults over 20, whether they had hepatic steatosis or not, were enrolled in the study after agreeing to participate following a full explanation of the study details. Individuals with the following conditions were excluded: (1) concurrent unstable cardiovascular diseases (including coronary artery disease, valvular heart disease, and acute stroke); (2) impaired activities of daily living; (3) history of cognitive impairment; (4) inability to cooperate with the examinations in this study; (5) refusal to participate. Participants signed a written informed consent after agreeing to participate in the study. The flowchart of the study is shown in Figure S1. This study was approved by the Research Ethics Committee of VGHTPE (protocol code: 2023‐08‐018BC, 2023‐08‐019BC).

Participants were evaluated by a questionnaire covering demographic data, health behaviors (smoking and alcohol consumption, physical activity), past medical history, and medication list. Additionally, they underwent physical measurement, blood tests, and VCTE.

### 2.2 Physical measurement

Blood pressure was measured twice at the clinic with a resting period of more than 20 minutes between measurements. The average blood pressure was calculated for analysis. Waist circumference (WC) was recorded at the midpoint between the lowest rib and the iliac crest at the end of expiration. Arm circumference was measured at the midpoint between the acromion of the scapula and the olecranon of the ulna when the arm and forearm were flexed to 90°. We measured the calf circumference at the widest position of the lower leg when the knee was flexed to 90°.

### 2.3 Blood tests

Participants received blood tests under a fasting status, including complete blood count and differential blood count (/μL), fasting plasma glucose (mg/dL), HbA1c (%), serum total cholesterol (mg/dL), triglyceride (TG, mg/dL), high density lipoprotein cholesterol (HDL‐C, mg/dL), low density lipoprotein cholesterol (LDL‐C, mg/dL), total insulin (uU/mL), creatinine (mg/dL), aspartate transaminase (AST, U/L), alanine aminotransferase (ALT, U/L), total bilirubin (mg/dL), albumin (g/dL), high sensitivity CRP (hsCRP, mg/dL), hepatitis B surface antigen (HBsAg, COI), hepatitis C antibody (Anti‐HCV, COI), LBP, and metabolites of SCFAs and tryptophan. The analyses of LBP were using commercial enzyme‐linked immunosorbent assay (ELISA) kits (MyBioSource, CA, USA). Other biochemical analyses were performed by automated analyzers in the central laboratory of Taipei Veterans General Hospital.

### 2.4 Evaluation of hepatic steatosis

Hepatic steatosis was mainly diagnosed by VCTE (FibroScan® 630 Expert), which was performed by experienced gastroenterologists. VCTE measured the CAP value. Referring to other Chinese studies and studies that include co‐existing liver diseases alongside fatty liver, a CAP value of ≥238 dB/m was regarded as hepatic steatosis and ≥ 292 dB/m was regarded as severe hepatic steatosis.^12^, ^13^ There were two participants whose VCTE was not available. For these two participants, hepatic steatosis was diagnosed in abdominal sonography performed by experienced gastroenterologists (TOSHIBA® i800 ultrasonic instrument, Toshiba, Tokyo, Japan) based on the attenuation imaging techniques.^14^

MAFLD was evaluated according to the international expert consensus in 2020.^15^ If participants had hepatic steatosis combined with type 2 diabetes mellitus, overweight, or obesity (body mass index, BMI ≥23 kg/m^2^), they were deemed as having MAFLD. Other participants of lean or normal weight who fulfilled at least two metabolic risk factors would also be diagnosed with MAFLD, including abdominal obesity, high blood pressure, low HDL‐C, hyperglycemia, insulin resistance, and high hsCRP level.^15^

### 2.5 Metabolic variables

Metabolic variables including homeostasis model assessment‐insulin resistance index (HOMA‐IR), TG, TG and glucose index (TyG), TG/HDL‐C ratio, TyG × BMI, TyG × WC, and visceral adipose index (VAI) were analyzed. HOMA‐IR was calculated as insulin (μU/ mL) × glucose (mg/dL)/405.^16^

TG/HDL‐C, TyG, TyG × BMI, and TyG × WC were proved to be valuable markers to recognize nonalcoholic fatty liver disease.^17^ TyG was calculated as ln[TG (mg/dL) × fasting plasma glucose (mg/dL)/2]. TyG × BMI and TyG × WC were obtained by multiplying the value of TyG with BMI and WC, respectively. VAI is a good indicator to visceral fat accumulation, which is calculated as^18^:

$$\mathrm{VAI}\left(\mathrm{men}\right)=\begin{array}{l} -267.93+0.68\times \mathrm{age}+0.03\times \mathrm{BMI}+4.00\times \mathrm{WC}+22.00\\ \times \log10\left(\mathrm{TG}\right)-16.32\times \mathrm{HDL}-C; \end{array}$$

$$\mathrm{VAI}\text{(women)}=\begin{array}{l} -187.32+1.71\times \mathrm{age}+4.23\times \mathrm{BMI}+1.12\times \mathrm{WC}\\ +\;39.76\times \log10\left(\mathrm{TG}\right)-11.66\times \mathrm{HDL}-C. \end{array}$$

### 2.6 Inflammatory variables

Neutrophil/HDL‐C ratio (NHR) was calculated by dividing the neutrophil count (×10^9^/L) by HDL‐C (mmol/L). AST/ALT ratio is the ratio of AST to ALT. hsCRP/Albumin ratio is the ratio of hsCRP (mg/dL) to albumin (g/dL). Other markers (hsCRP and LBP) representing inflammatory status were also included for analysis.

### 2.7 Metabolites of SCFAs and tryptophan

Metabolites of SCFAs and tryptophan were analyzed by the mass spectrometry (Shimadzu LC‐20Dxr; SCIEX Qtrap 5500). SCFAs metabolites comprised acetic acid, propionic acid, butyric acid, lactic acid, isobutyric acid, 2‐methylbutyric acid, isovaleric acid, valeric acid, 3‐methylvaleric acid, isocaproic acid, and caproic acid. The analysis of tryptophan includes the main metabolites from three metabolic pathways (tryptophan, kynurenine, 3‐hydroxyanthranilic acid, serotonin, quinolinic acid, indole‐3‐acetic acid, indole‐3‐propionic acid, indole‐3‐lactic acid, and indoxyl sulfate).

### 2.8 Statistical analyses and construction of prediction models

Categorical variables between severe and non‐severe hepatic steatosis groups were analyzed with chi‐squared tests, and continuous variables with Mann–Whitney *U* tests. To visualize correlations and group distributions of gut and clinical markers for severe vs. non‐severe hepatic steatosis, we generated heatmaps, correlation maps, and violin plots using Heatmapper (RRID:SCR_016974, http://heatmapper.ca/) and MetaboAnalyst version 6.0 (https://www.metaboanalyst.ca/). Variables that were significantly different between severe and non‐severe hepatic steatosis groups were selected for further analysis.

For construction of prediction models for severe hepatic steatosis, the continuous variables were categorized according to the optimal cut‐off values determined by receiver operating characteristic (ROC) curve analysis. These categorical variables were analyzed using multivariable logistic regression models and the *β* coefficients were regarded as weighting scores. The selection of variables to establish prediction models was based on the pathogenesis of MAFLD, including metabolic factors, inflammatory factors, and SCFAs and tryptophan metabolites. First, we selected the indicators for insulin resistance and metabolic disorders to construct the models. Subsequently, we included the markers for the gut–liver axis and SCFAs/tryptophan metabolites to create more precise models. The weight of the variables in the formula was determined by the statistical results of the multivariable logistic regression analyses, with severe hepatic steatosis as the dependent outcome variable. All these models were tested by ROC analyses. We further compared our models with another index proposed in previous literature, the hepatic steatosis index (HSI).^19^ All statistics were performed by SPSS 25.0. (IBM Corp., release 2017; IBM SPSS Statistics for. Windows, version 25.0, Armonk, NY, USA). A *p*‐value of <0.05 was considered statistically significant.

## 3 RESULTS

There were 83 participants recruited in this study (Table 1). The average age is 57.59 years and 68.67% of participants were male. 40 participants (48.2%) had severe hepatic steatosis. Compared to those without severe hepatic steatosis, individuals with severe hepatic steatosis were younger (mean age = 53.90 vs. 61.02 years, *p* = 0.01) and more obese (mean BMI = 30.07 vs. 28.04, *p* = 0.007; mean WC =100.06 vs. 95.84, *p* = 0.014). Individuals with severe hepatic steatosis also had higher levels of TG (mean level = 159.85 vs. 97.23, *p* < 0.001), ALT (mean level = 43.54 vs. 29.51, *p* < 0.001), arm circumference (mean level = 32.42 vs. 29.70, *p* = 0.001), calf circumference (mean level = 40.42 vs. 38.08, *p* = 0.004) and a lower level of HDL‐C (mean level = 44.28 vs. 53.37, *p* < 0.001). All of the participants with severe hepatic steatosis had MAFLD, while 74.42% of the participants with non‐severe hepatic steatosis had MAFLD (*p* < 0.001). There were no differences between participants with and without severe hepatic steatosis in sex, married status, education level, grouping of physical activity, smoking, alcohol use, maximal grip strength, past medical history including hypertension, hyperlipidemia, type 2 diabetes mellitus, cardiovascular disease, hepatitis B, and hepatitis C carrier status. The degree of liver stiffness is not significantly different between severe and non‐severe hepatic steatosis groups. Additionally, there were no differences in medication use, including antihypertensive drugs, diabetes medications, statins, fibrates, or any lipid‐lowering drugs.

**TABLE 1:** Basic characteristics of study population.

	Total (*n* = 83)	Severe hepatic steatosis^a^, *n* = 40 (48.2%)	Non‐severe hepatic steatosis, *n* = 43 (51.8%)	*p*
Age (years), *n* (%)	57.59 (13.61)	53.90 (12.57)	61.02 (13.79)	0.013
Male, *n* (%)	57 (68.67)	29 (72.50)	28 (65.12)	0.469
Married, *n* (%)	56 (67.47)	27 (67.50)	29 (67.44)	0.995
Education level, *n* (%)				
Below high school	30 (36.14)	12 (30.00)	18 (41.86)	0.261
College and above	53 (63.86)	28 (70.00)	25 (58.14)	
Physical activity^b^, *n* (%)				
Low	24 (28.92)	9 (22.50)	15 (34.88)	0.327
Moderate	45 (54.22)	25 (62.50)	20 (46.51)	
High	14 (16.87)	6 (15.00)	8 (18.60)	
Smoking, *n* (%)				
No	49 (59.04)	25 (62.50)	24 (55.81)	0.303
Current smoker	18 (21.69)	10 (25.00)	8 (18.60)	
Ex‐smoker	16 (19.28)	5 (12.50)	11 (25.58)	
Alcohol intake ≥140 g/week, *n* (%)	6 (7.23)	5 (11.63)	1 (2.50)	0.200
Past history, *n* (%)				
Hypertension	39 (46.99)	15 (37.50)	24 (55.81)	0.095
Hyperlipidemia	49 (59.04)	23 (57.50)	26 (60.47)	0.784
Type 2 diabetes mellitus	21 (25.30)	9 (22.50)	12 (27.91)	0.571
Cardiovascular disease^c^	9 (10.84)	4 (10.00)	5 (11.63)	0.995
Hepatitis B carrier	11 (18.97)	6 (20.69)	5 (17.24)	0.738
Hepatitis C carrier	4 (6.78)	1 (3.33)	3 (10.34)	0.353
MAFLD	72 (86.75)	40 (100)	32 (74.42)	<0.001
Liver stiffness measurement (LSM, kPa)	6.13 (2.50)	6.18 (2.16)	6.08 (2.82)	0.441
F3 (LSM ≥9.5)	8 (9.64)	3 (7.50)	5 (11.63)	0.714
F4 (LSM ≥12.5)	4 (4.82)	1 (2.50)	3 (6.98)	0.617
Medications				
Antihypertensive drugs	37 (44.58)	14 (35.00)	23 (53.49)	0.090
Diabetes drugs	21 (25.30)	8 (20)	13 (30.23)	0.284
All lipid drugs^d^	44 (53.01)	19 (47.50)	25 (58.14)	0.332
Statins	30 (36.59)	11 (28.21)	19 (44.19)	0.134
Fibrates	10 (12.20)	5 (12.82)	5 (11.63)	0.869
Blood tests, mean (SD)				
Creatinine (mg/dL)	0.89 (0.18)	0.88 (0.19)	0.91 (0.18)	0.469
Fasting plasma glucose (mg/dL)	110.57 (23.17)	112.08 (26.40)	109.14 (19.83)	0.930
HbA1c (%)	6.22 (0.82)	6.21 (0.80)	6.23 (0.86)	0.871
TG (mg/dL)	127.41 (67.24)	159.85 (73.82)	97.23 (42.81)	<0.001
HDL‐C (mg/dL)	48.99 (13.52)	44.28 (11.44)	53.37 (13.94)	<0.001
AST (U/L)	31.43 (22.26)	31.15 (24.52)	31.69 (20.24)	0.950
ALT (U/L)	36.18 (34.48)	43.54 (43.03)	29.51 (22.83)	<0.001
Albumin (g/dL)	4.53 (0.25)	4.49 (0.25)	4.58 (0.24)	0.110
Physical measurement, mean (SD)				
Systolic blood pressure (mmHg)	131.54 (15.28)	131.15 (15.21)	131.91 (15.52)	0.632
Diastolic blood pressure (mmHg)	76.36 (10.30)	78.43 (9.87)	74.44 (10.43)	0.075
Body mass index (kg/m^2^)	29.02 (4.64)	30.07 (4.15)	28.04 (4.90)	0.007
Waist circumferences (cm)	97.87 (11.41)	100.06 (9.11)	95.84 (12.97)	0.014
Arm circumference (cm)	31.01 (4.02)	32.42 (3.63)	29.70 (3.96)	0.001
Calf circumference (cm)	39.21 (4.05)	40.42 (3.18)	38.08 (4.46)	0.004
Maximal grip strength (kg)	36.87 (10.03)	37.53 (10.50)	36.25 (9.66)	0.678
Short physical performance battery	11.27 (1.20)	11.33 (1.27)	11.21 (1.15)	0.357

*Note*: Categorized variables were analyzed by chi‐squared tests and Fisher exact tests. Continuous variables were analyzed by Mann–Whitney *U* tests.

Abbreviations: ALT, alanine aminotransferase; AST, aspartate transaminase; HDL‐C, high density lipoprotein cholesterol; MAFLD, metabolic‐associated fatty liver disease; SD, standard deviation; TG, triglyceride.

^a^ Severe hepatic steatosis was diagnosed by abdominal sonography or vibration‐controlled transient elastography.

^b^ Physical activity was categorized according to the guidelines for data processing and analysis of the International Physical Activity Questionnaire (IPAQ).

^c^ Cardiovascular diseases indicated coronary artery disease, left ventricular hypertrophy, congestive heart failure, peripheral vascular disease, and stroke.

^d^ All lipid drugs included statins, fibrates, ezetimibe, niacin, and any other lipid drugs.

**FIGURE 1:**
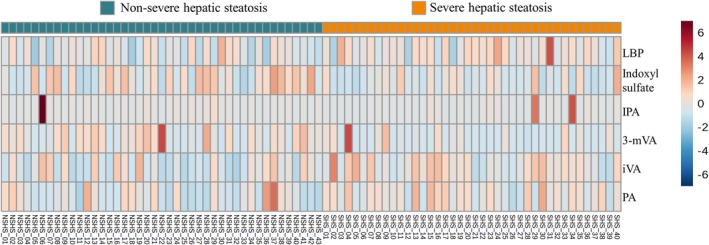
Heatmap of gut metabolites of study population. The concentrations of various metabolites were standardized through an autoscaling procedure. The red color represented higher concentrations, and the blue color represented lower concentrations. 3‐mVA, 3‐methylvaleric acid; IPA, indole‐3‐propionic acid; iVA, isovaleric acid; LBP, lipopolysaccharide binding protein; NSHS, non‐severe hepatic steatosis; PA, propionic acid; SHS, severe hepatic steatosis.

**FIGURE 2:**
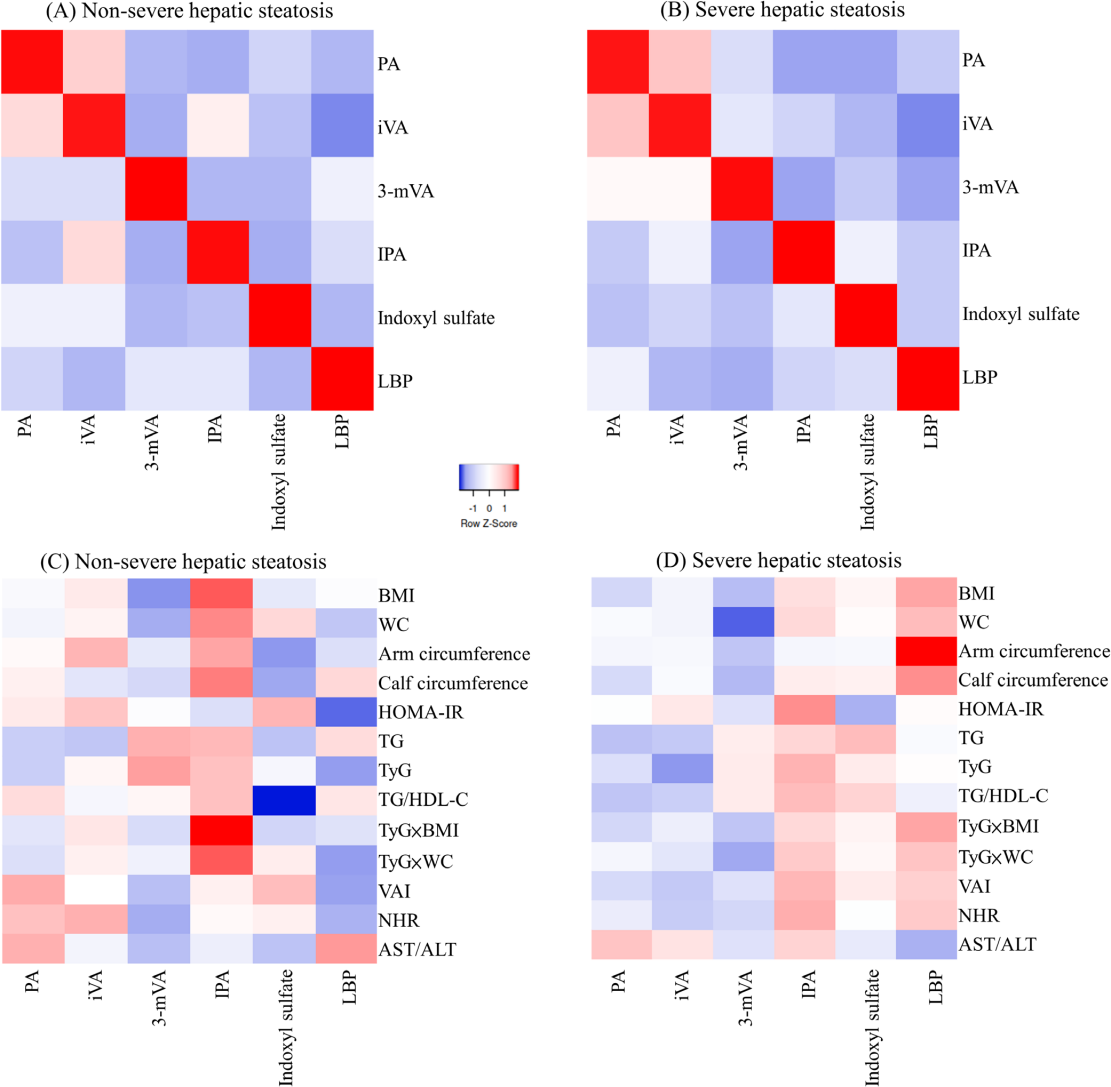
The correlation heatmap of *Z* scores for gut markers to gut markers (A, B) and gut markers to clinical markers (C, D), stratified by severe and non‐severe hepatic steatosis groups. 3‐mVA, 3‐methylvaleric acid; ALT, alanine aminotransferase; AST, aspartate transaminase; BMI, body mass index; HDL‐C, high density lipoprotein cholesterol; HOMA‐IR, Homeostasis Model Assessment‐Insulin Resistance index; IPA, indole‐3‐propionic acid; iVA, isovaleric acid; LBP, lipopolysaccharide binding protein; NHR, neutrophil/HDL‐C ratio; PA, propionic acid; TG, triglyceride; TyG, triglyceride‐glucose index; VAI, visceral adipose index; WC, waist circumferences.

Participants with severe hepatic steatosis had significantly higher levels of all metabolic variables including HOMA‐IR, TG/HDL‐C ratio, TyG, TyG × BMI, TyG × WC, and VAI (all *p* < 0.01, Table 2), higher levels of NHR, LBP, and a lower level of AST/ALT ratio. For SCFAs and tryptophan metabolites, severe hepatic steatosis group exhibited higher expression of propionic acid (mean level = 2.13 vs. 1.75, *p* = 0.022) and isovaleric acid (mean level = 0.48 vs. 0.37, *p* = 0.008), and lower expression of 3‐methylvaleric acid (mean level = 0.02 vs. 0.04, *p* = 0.001), indole‐3‐propionic acid (mean level = 1359.31 vs. 1452.34, *p* = 0.035), and indoxyl sulfate (mean level = 4313.06 vs. 5982.85, *p* = 0.039). For a more detailed expression of the SCFAs and tryptophan metabolites among different groups of participants with MAFLD, the comparison of SCFAs and tryptophan metabolites among three degrees of hepatic steatosis in MAFLD patients is provided in Table S1. Figure 1. visually illustrates the distribution of the magnitude of the relative amounts of metabolites between the severe and non‐severe hepatic steatosis groups. Severe hepatic steatosis group had higher levels of LBP, isovaleric acid, propionic acid and lower levels of indoxyl sulfate and 3‐methylvaleric acid. Figure 2. displays the correlation heatmap of *Z* scores for SCFAs/tryptophan metabolites and clinical markers. The negative correlation between LBP and 3‐methylvaleric acid was stronger in the severe hepatic steatosis group than in the non‐severe hepatic steatosis group (Figure 2A,B). In the severe hepatic steatosis group, LBP exhibited predominantly positive relationships with obesity markers (arm circumference, calf circumference, BMI) and the insulin resistance marker (TyG‐BMI) (Figure 2D).

**TABLE 2:** Metabolic variables, inflammatory variables, and metabolites of short chain fatty acid and tryptophan of study population.

	Total (*n* = 83)	Severe hepatic steatosis^a^, *n* = 40 (48.2%)	Non‐severe hepatic steatosis, *n* = 43 (51.8%)	*p*
Metabolic variables, mean (SD)				
HOMA‐IR	4.38 (3.71)	4.95 (3.03)	3.83 (4.22)	0.007
TG/HDL‐C ratio	2.93 (1.94)	3.95 (2.14)	1.98 (1.08)	<0.001
TyG^b^	8.72 (0.52)	8.98 (0.48)	8.48 (0.45)	<0.001
TyG × BMI	254.08 (46.83)	270.66 (44.74)	238.28 (43.63)	<0.001
TyG × Waist circumferences	855.43 (122.96)	899.99 (110.29)	812.99 (120.49)	<0.001
Visceral Adipose Index	172.46 (49.19)	187.23 (39.82)	158.72 (53.39)	0.001
Inflammatory variables, mean (SD)				
Neutrophil/HDL‐C ratio	1.59 (0.69)	1.78 (0.79)	1.41 (0.52)	0.023
AST/ALT ratio	1.10 (0.91)	0.79 (0.25)	1.38 (1.18)	<0.001
hsCRP/Albumin ratio	0.06 (0.09)	0.05 (0.04)	0.07 (0.11)	0.875
hsCRP (mg/dL)	0.39 (0.49)	0.35 (0.48)	0.42 (0.50)	0.524
Lipopolysaccharide binding protein (ng/mL)	135.60 (62.62)	152.15 (67.27)	120.20 (54.31)	0.025
Short chain fatty acid metabolites (μM), mean (SD)				
Lactic acid	4111.15 (1618.60)	4067.88 (1635.65)	4151.41 (1620.88)	0.771
Acetic acid	37.80 (16.40)	37.02 (18.83)	38.53 (13.96)	0.312
Propionic acid	1.94 (1.00)	2.13 (0.87)	1.75 (1.09)	0.022
Isobutyric acid	0.48 (0.16)	0.48 (0.12)	0.48 (0.19)	0.547
Butyric acid	0.49 (0.37)	0.54 (0.39)	0.45 (0.35)	0.146
2‐methylbutyric acid	0.41 (0.15)	0.40 (0.10)	0.41 (0.18)	0.655
Valeric acid	0.085 (0.084)	0.09 (0.08)	0.08 (0.09)	0.176
Isovaleric acid	0.42 (0.21)	0.48 (0.20)	0.37 (0.20)	0.008
Isocaproic acid	0.15 (0.10)	0.16 (0.10)	0.13 (0.10)	0.103
Caproic acid	0.44 (0.26)	0.42 (0.24)	0.45 (0.27)	0.423
3‐methylvaleric acid	0.03 (0.030)	0.02 (0.03)	0.04 (0.03)	0.001
Tryptophan metabolites (nM), mean (SD)				
Tryptophan	49,394.35 (7609.24)	50,996.71 (8058.00)	47,903.78 (6930.39)	0.186
Kynurenine pathway				
Kynurenine	1316.03 (409.35)	1273.97 (342.45)	1355.16 (463.70)	0.642
3‐Hydroxyanthranilic acid	17.06 (11.31)	19.84 (11.19)	16.91 (11.91)	0.103
Quinolinic acid	475.06 (185.40)	450.03 (153.96)	498.35 (209.64)	0.348
Indole pathway				
Indole‐3‐acetic acid	1971.13 (1243.89)	2023.28 (1367.19)	1922.61 (1131.44)	0.993
Indole‐3‐propionic acid	1408.09 (3907.42)	1359.31 (3528.45)	1452.34 (4264.44)	0.035
Indole‐3‐lactic acid	578.70 (284.25)	560.21 (282.51)	595.90 (288.11)	0.367
Indoxyl sulfate	5178.13 (3121.98)	4313.06 (2489.11)	5982.85 (3449.72)	0.039
Serotonin pathway				
Serotonin	774.80 (438.36)	818.38 (473.39)	734.25 (404.46)	0.433

*Note*: Analyses were performed by Mann–Whitney *U* tests.

Abbreviations: ALT, alanine aminotransferase; AST, aspartate transaminase; BMI, body mass index; HDL‐C, high density lipoprotein cholesterol; HOMA‐IR, Homeostasis Model Assessment‐Insulin Resistance index; hsCRP, high‐sensitivity C reactive protein; SD, standard deviation; TG, triglyceride; TyG, triglyceride‐glucose index; WC, waist circumference.

^a^ Severe hepatic steatosis was diagnosed by abdominal sonography or vibration‐controlled transient elastography.

^b^ TyG = ln [TG (mg/dL) × fasting plasma glucose (mg/dL)/2].

We selected some variables to construct prediction models for severe hepatic steatosis. Specifically, we chose one variable from the numerous markers of physical measurements and two variables from the numerous metabolic and inflammatory markers to reduce collinearity. We attempted various combinations of these factors to achieve maximal predictive power (Table 3). Initially, we selected variables relevant to inflammation, insulin resistance, and metabolic disorders to construct models (models 1 and 2). Then, we added LBP and 3‐methylvaleric acid as these two markers show a more reasonable directionality with other clinical variables in the correlation heatmap (Figure 2D) within the severe hepatic steatosis group (models 3 and 4). Finally, we further included other metabolites (propionic acid, isovaleric acid, indole‐3‐propionic acid, and indoxyl sulfate) to determine if the predictive power could be enhanced (models 5 and 6).

**TABLE 3:** Prediction models were developed based on the statistical results of multivariable logistic regression analysis for severe hepatic steatosis.

Models	Variables	*β*	*p*	OR (95% CI)
Model 1	Waist circumferences >103.5	1.11	0.117	3.02 (0.76–12.06)
	TyG >8.88	1.99	0.001	7.29 (2.24–23.76)
	AST/ALT ratio <0.98	1.63	0.004	5.10 (1.66–15.67)
	Constant	−1.98	<0.001	
Predictive scores = 1.11 (if Waist circumferences >103.5) + 1.99 (if TyG >8.88) + 1.63 (if AST/ALT ratio <0.98) −1.98				
Model 2	Arm circumference >32.75	0.83	0.215	2.23 (0.62–8.44)
	TG/HDL‐C >3.04	2.20	<0.001	9.04 (2.72–30.00)
	AST/ALT ratio <0.98	1.63	0.006	5.10 (1.60–16.22)
	Constant	−2.06	<0.001	
Predictive scores = 0.83 (if Arm circumference >32.75) + 2.20 (if TG/HDL >3.04) + 1.63 (if AST/ALT ratio <0.98) −2.06				
Model 3	Waist circumferences >103.5	1.09	0.170	2.97 (0.64–13.81)
	TyG >8.88	2.37	0.001	10.71 (2.62–43.83)
	AST/ALT ratio <0.98	1.60	0.014	4.93 (1.39–17.49)
	LBP >141.53	1.49	0.030	4.41 (1.20–16.29)
	3‐mVA <0.012	1.50	0.020	4.50 (1.25–16.10)
	Constant	−3.56	<0.001	
Predictive scores = 1.09 (if Waist circumferences >103.5) + 2.37 (if TyG >8.88) + 1.60 (if AST/ALT ratio <0.98) + 1.49 (if LBP > 141.53) + 1.50 (if 3‐mVA <0.012) −3.56				
Model 4	Arm circumference > 32.75	0.47	0.536	1.59 (0.36–6.97)
	TG/HDL‐C > 3.04	2.34	0.001	10.38 (2.71–39.71)
	AST/ALT ratio <0.98	1.65	0.012	5.18 (1.44–18.61)
	LBP >141.53	0.83	0.183	2.30 (0.68–7.84)
	3‐mVA <0.012	1.64	0.013	5.14 (1.42–18.58)
	Constant	−3.23	<0.001	
Predictive scores = 0.47 (if Arm circumference > 32.75) + 2.34 (if TG/HDL‐C > 3.04) + 1.65 (if AST/ALT ratio <0.98) + 0.83 (if LBP > 141.525) + 1.64 (if 3‐mVA <0.0115) −3.23				
Model 5	Waist circumferences >103.5	0.86	0.405	2.36 (0.31–17.72)
	TyG >8.88	3.12	0.002	22.64 (3.24–158.12)
	AST/ALT ratio <0.98	0.88	0.302	2.40 (0.46–12.67)
	LBP > 141.53	1.73	0.063	5.65 (0.91–34.95)
	3‐mVA <0.012	2.38	0.017	10.85 (1.54–76.55)
	PA >1.30	1.10	0.276	3.01 (0.42–21.74)
	iVA >0.29	3.24	0.012	25.58 (2.03–323.13)
	IPA < 744.68	1.70	0.072	5.48 (0.86–34.92)
	Indoxyl sulfate <5982.06	1.54	0.101	4.69 (0.74–29.67)
	Constant	−9.17	<0.001	
Predictive scores = 0.86 (if Waist circumferences >103.5) + 3.12 (if TyG >8.88) + 0.88 (if AST/ALT ratio <0.98) + 1.73 (if LBP > 141.53) + 2.38 (if 3‐mVA <0.012) + 1.10 (if PA >1.30) + 3.24 (if iVA >0.29) + 1.70 (if IPA < 744.68) + 1.54 (if Indoxyl sulfate <5982.06) −9.17				
Model 6	Arm circumference >32.75	0.24	0.796	1.27 (0.21–7.68)
	TG/HDL‐C > 3.04	2.34	0.003	10.35 (2.23–48.05)
	AST/ALT ratio <0.98	1.23	0.142	3.41 (0.66–17.55)
	LBP >141.53	1.08	0.178	2.95 (0.61–14.24)
	3‐mVA <0.012	2.24	0.011	9.36 (1.66–52.89)
	PA >1.30	0.86	0.344	2.36 (0.40–14.01)
	iVA >0.29	2.08	0.038	8.00 (1.13–56.83)
	IPA <744.68	1.61	0.060	5.02 (0.93–27.03)
	Indoxyl sulfate <5982.06	1.07	0.178	2.92 (0.61–13.86)
	Constant	−7.23	<0.001	
Predictive scores = 0.24 (if Arm circumference > 32.75) + 2.34 (if TG/HDL >3.04) + 1.23 (if AST/ALT ratio < 0.98) + 1.08 (if LBP > 141.53) + 2.24 (if 3‐mVA <0.012) + 0.86 (if PA >1.30) + 2.08 (if iVA >0.29) + 1.61 (if IPA < 744.68) + 1.07 (if Indoxyl sulfate <5982.06) −7.23				

Abbreviations: 3‐mVA, 3‐methylvaleric acid; ALT, alanine aminotransferase; AST, aspartate transaminase; HDL‐C, high density lipoprotein cholesterol; IPA, indole‐3‐propionic acid; iVA, isovaleric acid; LBP, lipopolysaccharide binding protein; PA, propionic acid; TG, triglyceride; TyG, triglyceride‐glucose index.

**FIGURE 3:**
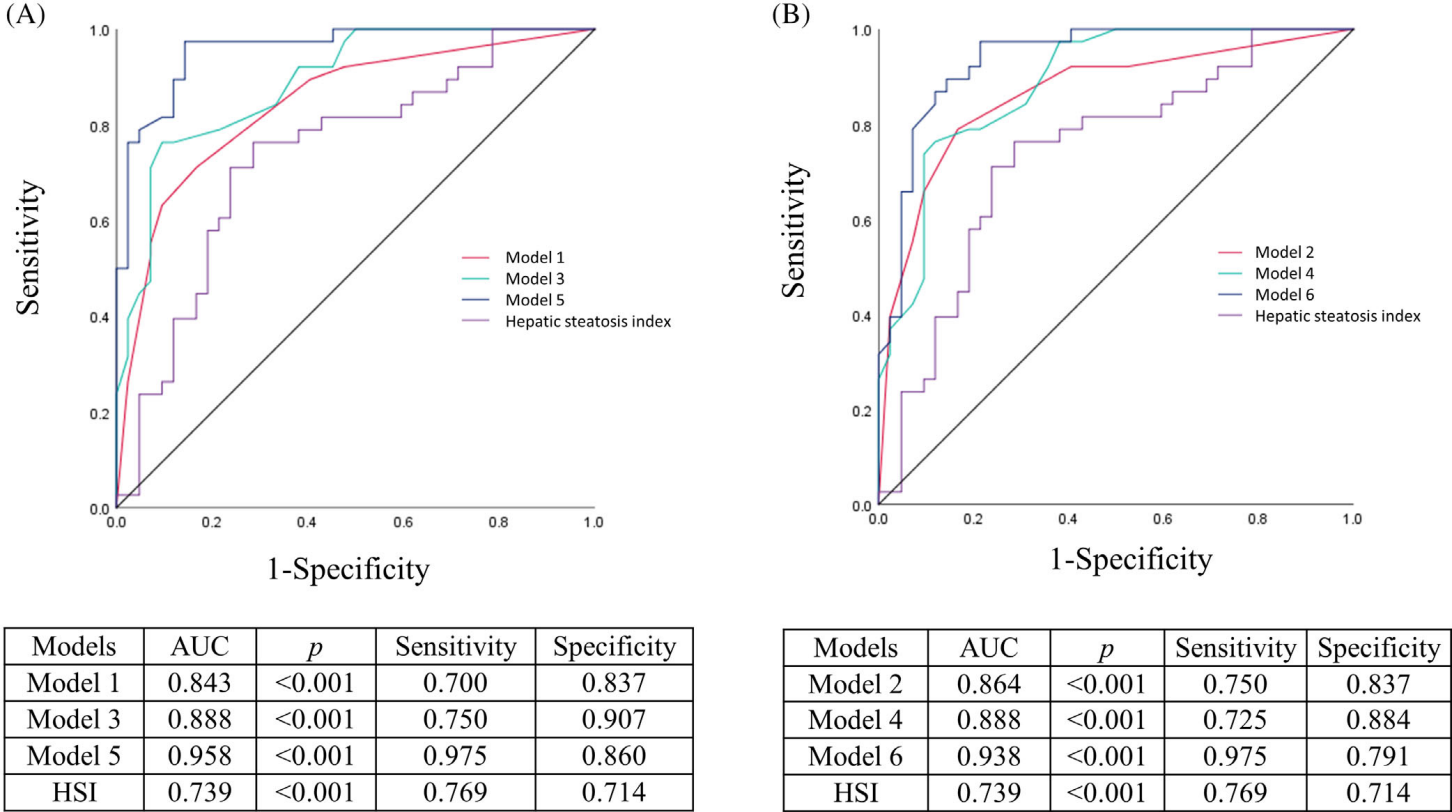
The ROC analyses of predictive models of severe hepatic steatosis. AUC, area under curve; HSI, hepatic steatosis index.

For the ROC analyses, models 1 and 2 achieved area under the curve (AUC) values of 0.843 and 0.864, respectively (Figure 3, *p* < 0.001). After adding the LBP and 3‐methylvaleric acid, the AUC increased to 0.888 and 0.888 for models 3 and 4, respectively (*p* < 0.001). The predictive capacity of severe hepatic steatosis could be further increased to AUC = 0.958 and 0.938 for models 5 and 6 after adding more variables of SCFAs/tryptophan metabolites (*p* < 0.001). In comparison, the AUC of the HSI is lower than that of the current models (AUC = 0.739).

## 4 DISCUSSION

This study found propionic acid, isovaleric acid, 3‐methylvaleric acid, indole‐3‐propionic acid, and indoxyl sulfate are important SCFAs/tryptophan metabolites correlated well with severe hepatic steatosis. Prediction models incorporating these variables demonstrated good predictive capacity for severe hepatic steatosis. To our knowledge, this study is the first to include markers of the gut–liver axis and SCFAs/tryptophan metabolites in the prediction of severe hepatic steatosis.

Thing et al. had analyzed 100 MAFLD patients' plasma and found MAFLD patients had higher levels of propionate, formate, valerate but lower level of acetate.^6^ Tsai et al. analyzed 259 Taiwanese patients with type 2 diabetes mellitus. They found lower levels of serum formate, isobutyrate, and methylbutyrate in the moderate and severe NAFLD groups compared with the no or mild NAFLD group. They also indicated that isobutyrate and methylbutyrate were closely related to the severity of NAFLD.^20^ Xiong et al. ever reported that the levels of plasma acetate, butyrate, and propionate in 71 patients with NAFLD decreased as the severity of NAFLD progressed to nonalcoholic steatohepatitis and cirrhosis. They also found that the levels of plasma acetate, butyrate, and propionate were higher in the NAFLD group than in the healthy control group, although this difference was not statistically significant.^21^ These results were partly consistent with our findings that patients with severe hepatic steatosis exhibited higher concentrations of serum propionic acid and isovaleric acid, and a lower concentration of 3‐methylvaleric acid.

Propionic acid can have beneficial effects on health at appropriate concentration, including potentially reducing fatty acid accumulation in the liver and improving insulin sensitivity.^22^ Excessive propionic acid activated microglia, suggesting a neuroinflammatory process.^23^ It is unknown how much serum concentration of propionic acid is beneficial to the liver, and further research is needed to clarify this question. Studies about isovaleric acid, 3‐methylvaleric acid and MAFLD are lacking. A mouse study demonstrated that the increase in fecal isovaleric acid stimulated hepatic insulin resistance.^24^ A study ever reported that 5‐aryl‐3‐methylvaleric acid derivatives, which share a similar structure with 3‐methylvaleric acid, have the effect of lowering serum cholesterol and triglycerides.^25^ Our results may be consistent with the literature, as we found a higher mean serum concentration of isovaleric acid and a lower mean serum concentration of 3‐methylvaleric acid among participants with severe hepatic steatosis. More studies are needed to clarify the roles of isovaleric acid and 3‐methylvaleric acid in MAFLD.

In our study, indole pathway products, indole‐3‐propionic acid and indoxyl sulfate, were lower in the severe hepatic steatosis group. Indole‐3‐propionic acid benefits metabolic health by lowering blood glucose, enhancing insulin sensitivity, reducing hepatic lipid synthesis, maintaining gut barrier integrity, and correcting gut dysbiosis.^26^ Indole‐3‐propionic acid has also been reported to be lower in obese patients with hepatic lobular inflammation compared to those without such inflammation.^27^ Indoxyl sulfate is also a metabolite derived from the indole pathway. Lin et al. ever reported that the level of indoxyl sulfate decreases as the severity of liver cirrhosis in patients progresses.^28^ These studies echo the findings of this study, indicating that indole‐3‐propionic acid and indoxyl sulfate can serve as potential biomarkers for severe hepatic steatosis.

There are some limitations in this study. First, the sample size is small and validation of the prediction models is lacking. The cross‐sectional design does not allow for causal inference of the relevant variables and the development of MAFLD. Further longitudinal study with a large sample size is needed. Second, we classified participants into severe and non‐severe hepatic steatosis groups. The non‐severe hepatic steatosis group is heterogeneous, which may bias the results. Nevertheless, the heterogeneity of the control group tends to lead to null effects,^29^ which means that the variables correlated with severe hepatic steatosis identified in this study should be reliable. Third, histology and MRI are more accurate tools for diagnosing hepatic steatosis. However, these data were unavailable in our study population. We used VCTE because of its good accuracy in detecting hepatic steatosis.^9^ Fourth, we included patients with viral hepatitis and alcoholic liver disease to reflect a real‐world scenario. There was no difference in their proportions between groups, and VCTE remained a strong predictor of liver steatosis despite other liver diseases.^30^ Fifth, two participants lacked VCTE data, so sonography was used, which could lead to misclassification. However, ultrasonography has good sensitivity and specificity for moderate to severe fatty liver compared to histology.^31^, ^32^ These two cases were identified as moderate fatty liver, making misclassification unlikely. Finally, data on the use of probiotics, prebiotics, synbiotics, and postbiotics is unavailable. Future studies should consider the effects of these substances to better understand their role in gut metabolites and MAFLD.

Despite these limitations, this study employed SCFAs and tryptophan metabolites relevant to the pathogenesis of MAFLD to predict severe hepatic steatosis, presenting a novel approach. Combined with other clinical markers, we found propionic acid, isovaleric acid, 3‐methylvaleric acid, indole‐3‐propionic acid, and indoxyl sulfate are valuable in predicting severe hepatic steatosis. Future research can further explore the roles of these markers in MAFLD pathogenesis in vivo and in vitro.

In conclusion, we establish integrated models comprising SCFAs/tryptophan metabolites and other clinical variables, showing good predictivity of severe hepatic steatosis. Studies are warranted to deeply investigate the impact of SCFAs and tryptophan metabolites on the pathogenesis of MAFLD.

## CONFLICT OF INTEREST STATEMENT

The authors declare that there are no conflicts of interest.

## Supporting information


**Figure S1.** Recruitment flowchart of the study population.

**Figure S2.** The violin plot of gut markers grouped by severe and non‐severe hepatic steatosis.

**Table S1.** The comparison of SCFAs and tryptophan metabolites among different groups of participants with MAFLD (*n* = 72).

## Data Availability

The data from this study is restricted to use solely for this research and cannot be publicly disclosed. Interested researchers may contact the corresponding author to obtain the data.
