# Supplementary figures and images for: Models incorporating physical, laboratory and gut metabolite markers can be used to predict severe hepatic steatosis in MAFLD patients
Source: Kaohsiung J Med Sci. 2024 Nov 4;40(12):1095–105. doi: 10.1002/kjm2.12904 (PMC11618486; doi:10.1002/kjm2.12904)

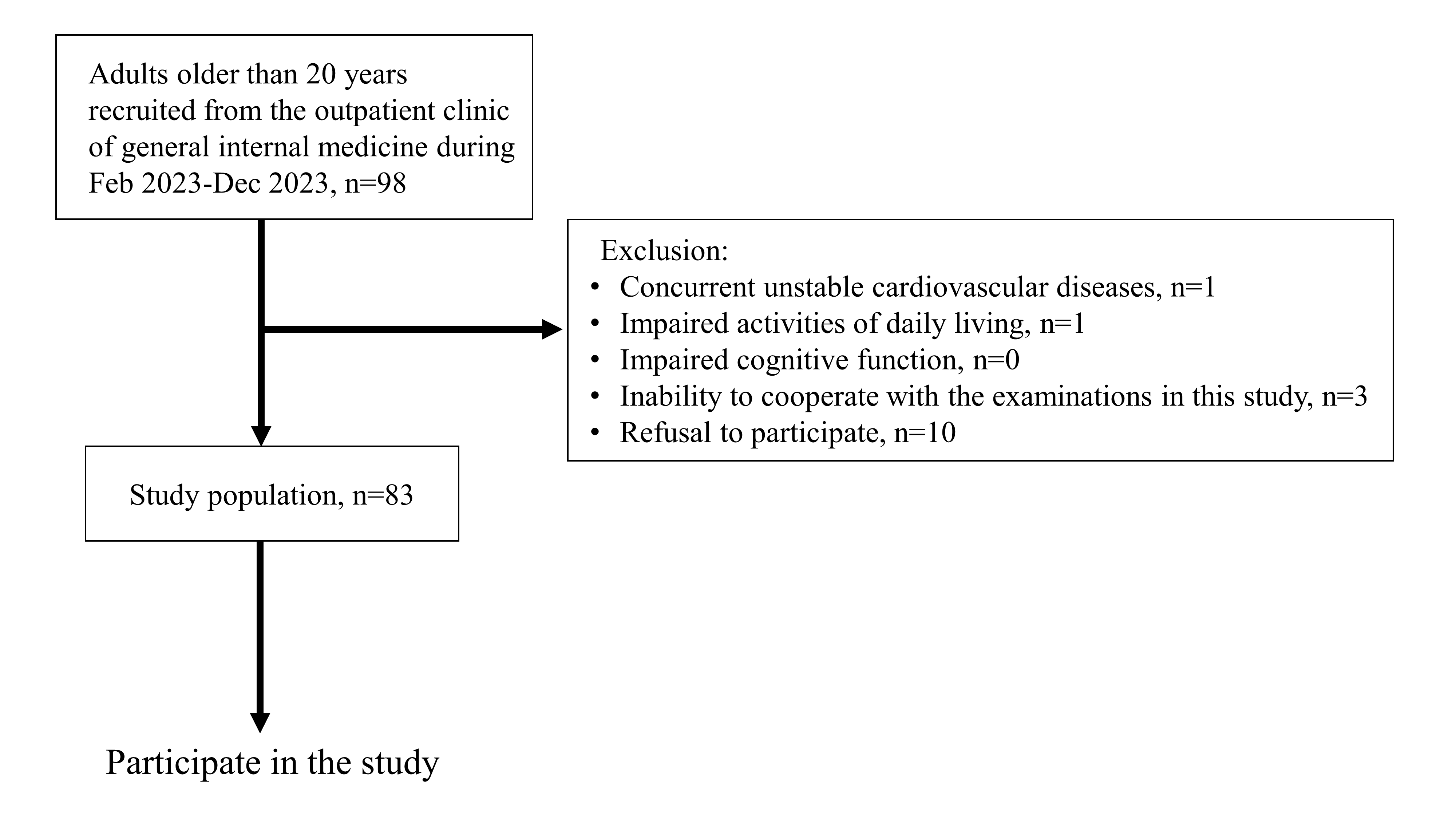

Supplement: Supplementary file 1 — Figure S1. Recruitment flowchart of the study population. [file KJM2-40-1095-s002.tif]

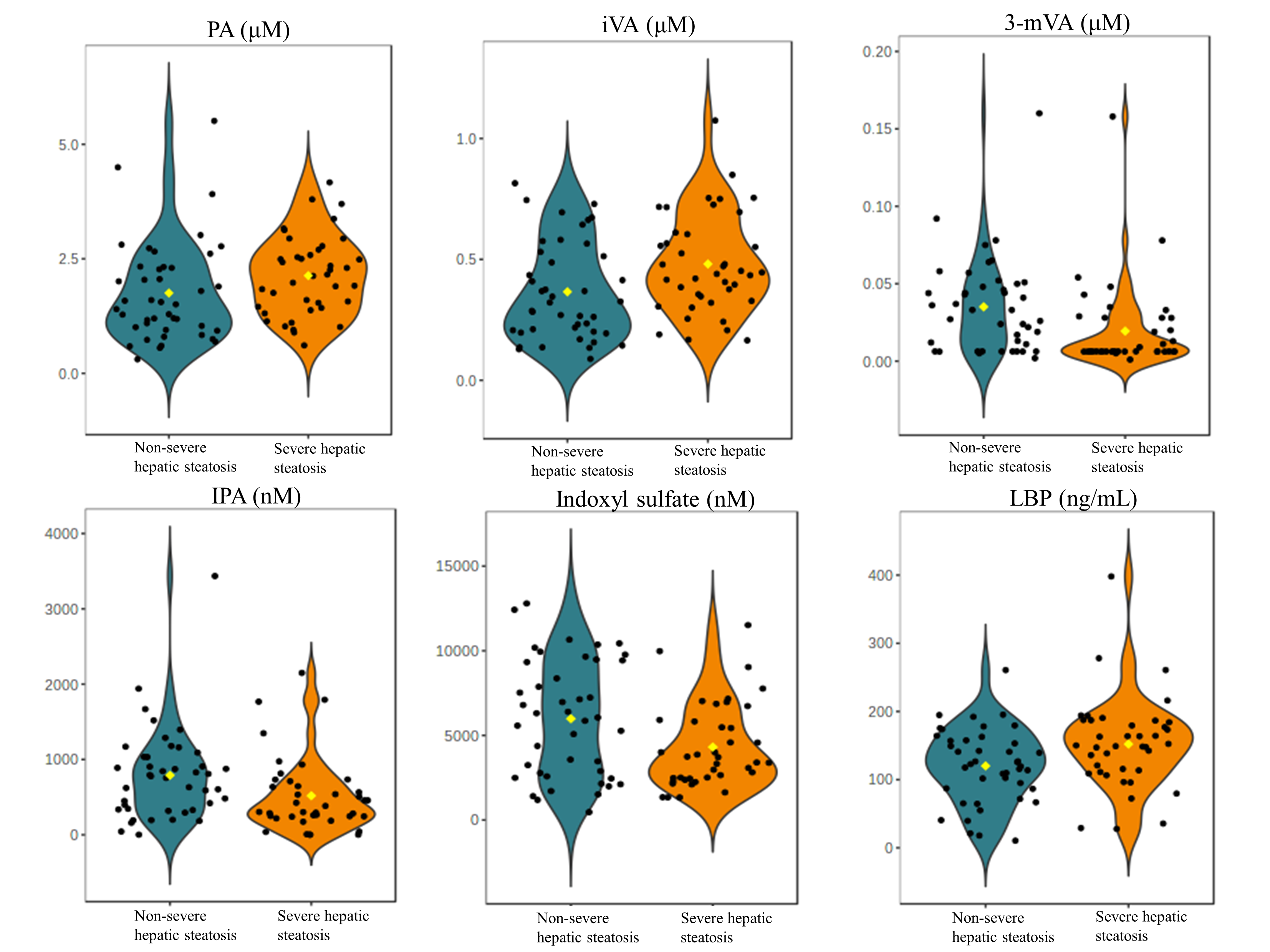

Supplement: Supplementary file 2 — Figure S2. The violin plot of gut markers grouped by severe and non‐severe hepatic steatosis. [file KJM2-40-1095-s003.tif]
