# Supplementary material for: Models incorporating physical, laboratory and gut metabolite markers can be used to predict severe hepatic steatosis in MAFLD patients
Source: Kaohsiung J Med Sci. 2024 Nov 4;40(12):1095–105. doi: 10.1002/kjm2.12904 (PMC11618486; doi:10.1002/kjm2.12904)
Supplement: Supplementary file 3 — Table S1. The comparison of SCFAs and tryptophan metabolites among different groups of participants with MAFLD (n = 72). [file KJM2-40-1095-s001.docx]

**Supplementary TABLE 1** The comparison of SCFAs and tryptophan metabolites among different groups of participants with MAFLD (n=72).

|  | Mild hepatic steatosis | Moderate hepatic steatosis | Severe hepatic steatosis | *p* |
| --- | --- | --- | --- | --- |
|  | n=10 (13.89%) | n=22 (30.56%) | n=40 (55.56%) |  |
| SCFAs metabolites |  |  |  |  |
| lactic acid (LA) | 4132.6 (1322.13) | 4110.38 (1724.70) | 4067.88 (1635.65) | 0.88 |
| acetic acid (AA) | 36.82 (6.14) | 38.75 (16.45) | 37.02 (18.83) | 0.775 |
| propionic acid (PA) | 1.59 (0.72) | 1.65 (0.90) | 2.13 (0.87) | 0.054 |
| isobutyric acid (iBA) | 0.46 (0.12) | 0.47 (0.17) | 0.48 (0.12) | 0.820 |
| butyric acid (BA) | 0.48 (0.31) | 0.40 (0.34) | 0.54 (0.39) | 0.212 |
| 2-methylbutyric acid (2-mBA) | 0.41 (0.15) | 0.41 (0.19) | 0.40 (0.10) | 0.682 |
| valeric acid (VA) | 0.07 (0.06) | 0.07 (0.05) | 0.09 (0.08) | 0.433 |
| isovaleric acid (iVA) | 0.37 (0.21) | 0.36 (0.20) | 0.48 (0.20) | 0.040 |
| isocaproic acid (iCA) | 0.08 (0.05) | 0.15 (0.12) | 0.16 (0.10) | 0.011 |
| caproic acid (CA) | 0.44 (0.25) | 0.44 (0.32) | 0.42 (0.24) | 0.908 |
| 3-methylvaleric acid (3-mVA) | 0.04 (0.02) | 0.05 (0.04) | 0.03 (0.04) | 0.119 |
| Tryptophan metabolites |  |  |  |  |
| tryptophan (Trp) | 49454.3 (3446.09) | 47568.98 (8426.70) | 50996.71 (8058.01) | 0.586 |
| kynurenine (Kyn) | 1486.88 (646.99) | 1309.54 (408.69) | 1273.97 (342.45) | 0.779 |
| 3-Hydroxyanthranilic acid (HAA) | 17.80 (16.83) | 16.87 (10.09) | 19.84 (11.19) | 0.520 |
| Serotonin | 712.6 (318.91) | 806.32 (447.08) | 818.38 (473.39) | 0.914 |
| Quinolinic acid (QA) | 519.67 (303.19) | 492.32 (159.16) | 450.03 (153.96) | 0.524 |
| Indole-3-acetic acid (IAA) | 1535.86 (768.18) | 1936.48 (1055.80) | 2023.28 (1367.19) | 0.614 |
| Indole-3-propionic acid (IPA) | 809.84 (496.87) | 2024.96 (5953.71) | 1359.31 (3528.45) | 0.225 |
| Indole-3-lactic acid (ILA) | 502.83 (132.67) | 586.60 (281.96) | 560.21 (282.51) | 0.700 |
| Indoxyl sulfate | 4182.87 (2580.20) | 5580.91 (3237.80) | 4313.06 (2489.11) | 0.268 |

Analyses were performed by Kruskal Wallis tests.

The categorization of steatosis was determined by the CAP value. A CAP value of ≥ 238 dB/m and <259 dB/m was regarded as mild hepatic steatosis; ≥ 259 dB/m and <292 dB/m was regarded as moderate hepatic steatosis; and ≥ 292 dB/m was regarded as severe hepatic steatosis.
